# Analysis of Factors Contributing to the Low Survival of Cervical Cancer Patients Undergoing Radiotherapy in Kenya

**DOI:** 10.1371/journal.pone.0078411

**Published:** 2013-10-30

**Authors:** Innocent O. Maranga, Lynne Hampson, Anthony W. Oliver, Anas Gamal, Peter Gichangi, Anselmy Opiyo, Catharine M. Holland, Ian N. Hampson

**Affiliations:** 1 University of Manchester, Viral Oncology, Research Floor, St Mary's Hospital, Manchester, United Kingdom; 2 Obstetrics and Gynaecology, University of Nairobi, Nairobi, Kenya; 3 Cancer Treatment Centre, Kenyatta National Hospital, Nairobi, Kenya; 4 Obstetrics and Gynaecology, Mansoura University Hospital, Cairo, Egypt; Baylor College of Medicine, United States of America

## Abstract

**Background:**

In contrast to the developed nations, invasive cervical cancer (ICC) is the most common womens malignancy in Kenya and many other locations in sub-Saharan Africa. However, studies on survival from this disease in this area of the world are severely restricted by lack of patient follow-up. We now report a prospective cohort study of ICC in Kenyan women analysing factors affecting tumour response and overall survival in patients undergoing radiotherapy.

**Methods and Findings:**

Between 2008 and 2010, 355 patients with histologically confirmed ICC were recruited at the Departments of Gynaecology and Radiotherapy at Kenyatta National Hospital (KNH). Structured questionnaires were completed recording socio-demographics, tumour response and overall survival following treatment with combinations of external beam radiation (EBRT), brachytherapy and adjuvant chemotherapy. Of the 355 patients, 42% (146) were lost to follow-up while 18% (64) died during the two year period. 80.5% of patients presented with advanced stage IIB disease or above, with only 6.7% of patients receiving optimal combined EBRT, brachytherapy and adjuvant chemotherapy. Kaplan Meier survival curves projected two year survival at <20%.

**Conclusion:**

Cervical cancer is preventable yet poverty, poor education, lack of cancer awareness coupled with an absence of regular screening programs, late patient presentation, sub-optimal diagnosis and treatments are major factors contributing to the alarmingly low survival rate of cervical cancer patients in Kenya. It is concluded that simple cost-effective changes in clinical practice could be introduced which would have a marked impact on patient survival in this setting.

## Introduction

Invasive cervical cancer (ICC) is the most common cause of cancer deaths in Africa accounting for 10.4%, which represents one in five of all cancer deaths in African women[1]. Indeed sub-Saharan Africa bears the highest global burden of this fatal yet entirely preventable disease [2]. Global ICC incidence estimates stand at 500,000 cases annually producing 300,000 deaths of which 85% occur in developing countries such as Kenya [3].

The incidence of ICC has plummeted in the developed countries over the last two decades mainly due to the implementation of national screening programs [4]. In these countries screening detects cancers at an early stage in the disease progression and cure rates are high. Equally they have better treatment outcomes for more advanced disease. However, in low resource countries, 80% of cervical cancer cases are very advanced at presentation and treatment outcomes are poor [5]. Indeed in many developing countries the ICC mortality to incidence ratio is high and often exceeds 0.5 [6]. It is a fact that five out of six women with cervical cancer live in developing countries, which possess only 5% of the global resources for cancer control [5]. Thus it is ironic that countries least equipped to treat cervical cancer have the largest burden of this disease[7].

There are few cytology screening programmes with coverage sufficient to have any impact on ICC in developing countries where standard radiotherapy treatment facilities are also severely limited. For most patients, lack of screening and treatment facilities combined with poverty, late presentation, diminished awareness of the preventable nature of ICC and a fatalistic attitude are all contributory factors [8], [9], [10]. Other concerns are poor follow-up, lack of trained personnel, unaffordable treatments in combination with socio-economic and cultural factors which all operate within an ill-structured health-care system.

In addition, cervical cancer has been classified as an AIDS-defining illness in women with HIV infection which is a recognized prognostic indicator of poor treatment outcome for ICC [11]. Indeed the bulk of global ICC and HIV/AIDS cases are found in developing countries such as Kenya.

Radiation therapy (RT), surgery and adjuvant chemotherapy remain standard treatment options for ICC with RT being the first line treatment although significant numbers of patients fail to respond [3]. Radical surgery and RT are both equally effective for early (1A1 and 2A) disease with the former being the treatment of choice for young women with 1A2, 1B1 or 2A disease. Women with 1A1 disease are treated with cone biopsy or simple hysterectomy whereas chemo-radiotherapy is the treatment of choice for 1B2 disease and above [12].

In the current study we sought to characterise a range of factors which may affect tumour response to various treatment options available to women attending for radiotherapy at Kenyatta National Hospital (KNH). By analysing such factors it may prove possible to implement simple cost-effective changes in practice which may improve the survival of these patients.

## Methods

The study was carried out between March 2008 and February 2010 where 355 consecutive patients with histologically verified ICC were recruited and followed up at KNH Departments of Radiotherapy and Gynaecology. KNH is the University of Nairobi's teaching and referral hospital with a 2000 bed capacity. The Departments of Obstetrics and Gynecology and Radiotherapy make up the largest ICC treatment centre in Kenya receiving patients from all over the country. Following recruitment a structured questionnaire was completed detailing the patients' socio-demographics, obstetric and gynaecological history and sexual history. Questionnaires were available in English (universal medium of teaching and communication in Kenya), Swahili (national language) or in local dialect as applicable. Patient visits and management were as per hospital protocol including: pre-radiation assessment, marking for external beam radiotherapy (EBRT), EBRT for ∼25 sessions and scheduled appointments. Pre-radiation assessment is standardized in the unit and entails complete physical examination, tests for haemoglobin, urea & electrolyte levels, chest x-ray, intravenous urogram (IVU) and examination under anesthesia for clinical staging according to the International Federation of Gynecology and Obstetrics 1995 (FIGO) system (see Table 1). Other radiological investigations like MRI, CT-Scan and ultra-sound scans were not routine due to cost-implications. Those who had low haemoglobin were transfused to at least 10 g/dL before commencing EBRT. The Unit encourages an open-door policy whereby patients can be seen outside these appointments.

**Table 1:** FIGO staging system for cervical carcinoma prior to the 2009 revision.

Stage	Findings
IA	Micro-invasive carcinoma.
IB:	Macroscopic Invasive cancer confined to the cervix
IIA	Tumour extending to upper third of vagina but not to the parametrium.
IIB	Tumour extending to the parametrium.
IIIA	Tumour involving the lower third vagina with no extension to the pelvic side wall
IIIB	Tumour extending to pelvic side wall and/or hydronephrosis or non-functioning kidney
IV A	Tumour involving adjacent pelvic organs i.e. bladder or rectum.
IV B	Extra-pelvic spread, e.g. metastasis to the liver, lungs etc

All patients in this study were assessed using the same pre-2009 staging system.

All the patients reported in this study received EBRT using a Cobalt 60 (Siemens or Theratron T280) machine, via parallel-opposed anterior and posterior fields (AP/ PA). The field sizes were adopted depending on the FIGO clinical stage of the disease. Patients in stage I–IIA were treated with 15×15 cm portals (at patient's surface) and 18×15 cm for stage IIB, III and IV. 94.9% patients (337/355) received a dose of 40–50 Gy to point A. Fractionation was 1.8–2.0 Gy tumor dose daily, 5 fractions per week for 5 weeks with 2 days rest from treatment during the weekend. Point A was defined as 2 cm above the external os and 2 cm lateral to the uterine canal. 8.2% (29/355) of patients received high dose fractions, 3.6–4.0 Gy as emergency radiotherapy to stop vaginal bleeding and were continued on regular fractions thereafter. Patients were assessed for eligibility for concurrent chemo/radiotherapy (CCRT) and those eligible were admitted. The qualifying criteria for chemotherapy included functional renal status, a haemoglobin level >10 g/dL and a sufficiently stable clinical condition to withstand treatment related complications. Chemotherapeutic agents used were cisplatin 50 mg/M^2^ on day one and 5fluorouracil 1000 mg/m2 IV on days 1–4 with a repeat cycle every 21 days. Following this treatment, patients were referred to neighbouring countries for brachytherapy since the equipment available at KNH was non-functional during this study.

As part of the standard Unit protocol, during the follow up visits, patients' symptoms were noted and a physical examination carried out using pelvic bimanual examination to assess tumour response to treatment. Any additional investigations such as ultra-sound scans, CT-scans, X-rays, MRI, and biopsy were optional due to cost implications. Thus, for the current study, tumor control was primarily documented by physical examination [13], [14] which was verified histologically whenever possible. Additionally, acute toxicity was documented using the Radiation Therapy Oncology Group and the European Organization for Research and Treatment of Cancer (RTOG/EORTC) Radiation Toxicity Grading [15]. Acute toxicity was defined as adverse events occurring during treatment and up to 90 days after completion of chemotherapy or radiotherapy.

### Statistical analysis

The main outcome measures were pelvic tumour control at 4–7 months from the last day of EBRT and overall survival following this or, where available, either brachytherapy or adjuvant chemotherapy. Data were entered and analyzed using SPSS version 16.0 (SPSS inc. Chicago, Illinois, USA). Comparison of means and proportion was done using Pearson's Chi-square tests, Fishers exact test and Student t-test where appropriate. Odds ratio (OR), adjusted OR (AOR) and the 95% Confidence intervals (CI) were used to measure strengths of associations.

Relative risk (RR) in univariate analysis and adjusted relative risk (ARR) on multivariate analysis were also computed. Cox regression multivariate analysis was used to estimate the hazard of pelvic tumor, while Kaplan-Meier statistical methods were used to calculate survival curves. A p-value (two-tailed test) of 0.05 was considered statistically significant. The study was approved by the Kenyatta National Hospital's Ethics and Research Committee (ERC), the University of Nairobi and the University of Manchester. All patients gave informed written consent to participate in this study.

## Results

### Age Distribution

The mean age was 49 (Range 21–94 years) with 28.2% of women aged between 40 and 49 yrs. The peak age for ICC incidence was 47 although this was 37 for HIV+ve Women.

### Awareness of Cervical Screening Procedures

Only 126 (35.5%) of patients with ICC had heard of a cervical screening. Similarly, only 54 (15.3%) patients had ever had a cervical smear; of which 9 never received their results.

### HIV Status and Whether in Receipt of Highly Active Antiretroviral Therapy (HAART)

Out of 355 patients, 189 (53.2%) had a HIV test prior to starting radiotherapy of which 52 (27.5%) were HIV+ve, while 137 (72.5%) were negative. Of the HIV+ve's 27 (52.0%) were on highly active antiretroviral therapy (HAART) whilst 25 (48.0%) were not.

### Haemoglobin (Hb) Status

At diagnosis 43.5% of patients had HB >10 g/dL, while 33.8% had between 8–10 g/dL and 22.7% had HB <8 g/dL. 37.0% of patients received a blood transfusion during the study period.

### Histological Type and Staging

**Figure 1:**
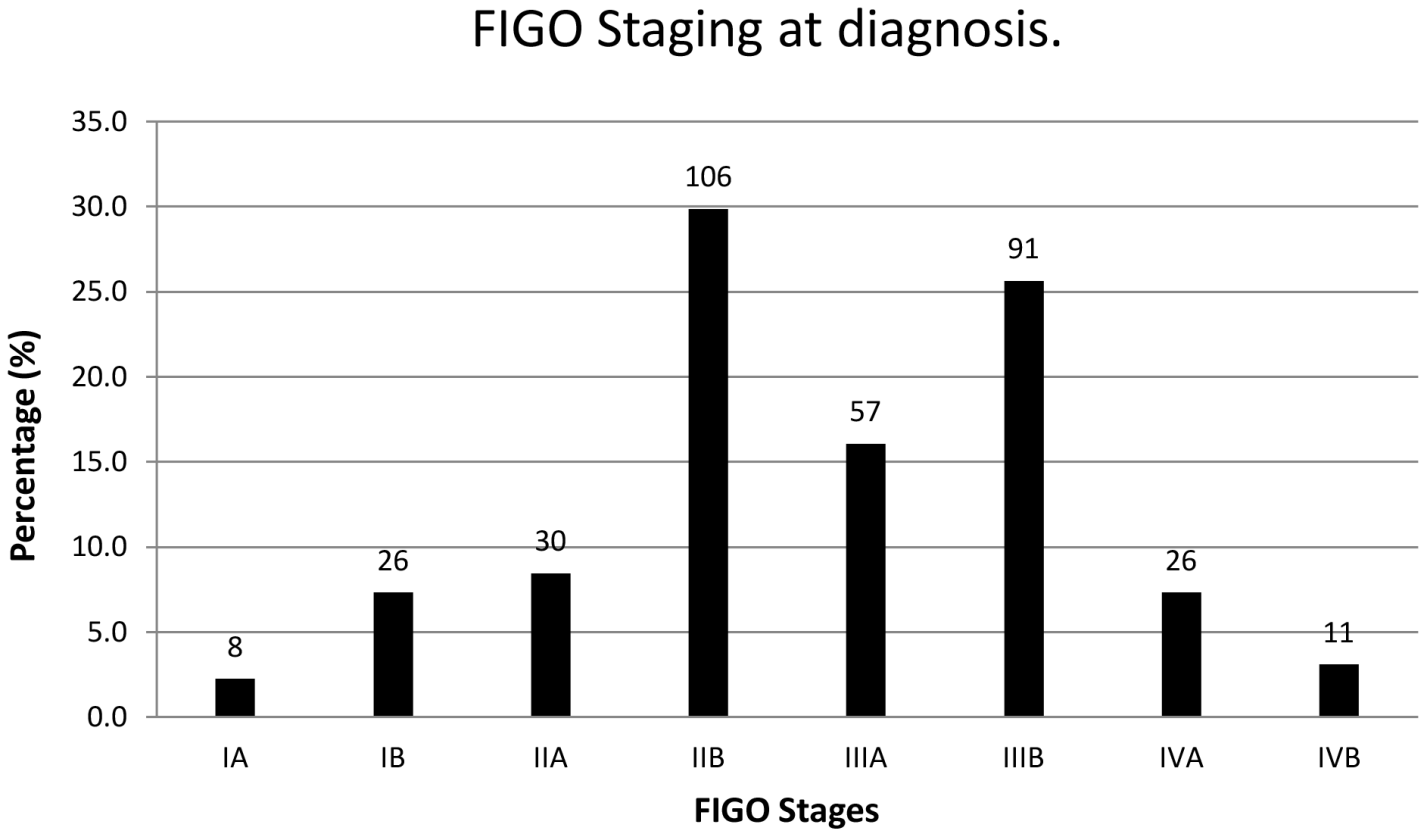
FIGO staging of disease at diagnosis. The numbers of patients is shown above each bar (n = 355) of which the majority (80.5%) presented late at stage 2B and above.

The most prevalent histological type of ICC was squamous cell carcinoma (SCC) (89.9%), followed by adenocarcinoma (AC) (5.6%). Two patients had anaplastic carcinoma, and another two had sarcoma of the cervix. Among those with SCC, most had moderately differentiated SCC (39.2%), with 32.0% and 21.3% having poorly differentiated and well differentiated disease respectively (Table 2). At the time of diagnosis the majority of patients (80.5%) presented with stage 2B disease or above as shown in Figure 1.

**Table 2:** Histological types of disease (n = 355).

Histological diagnosis	Frequency	%
SCC	318	89.5
Well Differentiated	68	21.3
Moderate	124	39.2
Poorly	102	32.0
Keratinizing	14	4.4
Large Cell non-keratinizing	9	2.8
Small cell	1	0.3
Anaplastic Carcinoma	2	0.6
Adenocarcinoma	20	5.6
Mixed Sq.	13	3.7
Sarcoma of Cervix	2	0.6

Squamous cell carcinoma was the commonest histological type with 89.5%, while adenocarcinomas accounted for 5.6%.

### Follow-Up

During the 2 year period out of the initial 355 patients, 146 (41.1%) patients were lost to follow-up. Of these 146 patients: 31 had a complete course of EBRT with no other treatments; 86 received partial courses of EBRT alone; 10 received a combination of partial EBRT and chemotherapy; 3 received partial chemotherapy alone; none had brachytherapy, while 16 received no definitive treatment at all. Therefore, among the 355 patients, 240 completed EBRT only.

Of those where follow up was available (n = 209), this had a mean duration of 16.8 months following commencement of treatment with a range of between 6 and 30 months. Out of these, 121 (57.9%) were diagnosed as having progressive disease while 64 (30.6%) patients died during the follow-up period. All the followed up patients received ∼25 sessions of EBRT (2 Gy per session) although 67% of them also received additional adjuvant therapies such as external boost radiotherapy (48.6%), brachytherapy (11.4%), chemotherapy (25.7%) and 37.9% used traditional herbal remedies. Only 6.7% of patients received the recommended treatment of combined EBRT, brachytherapy and chemotherapy with the majority receiving only EBRT (Table 3). The criterion for chemotherapy was met by 42% whereas 36% were ineligible and this could not be established in 22% of cases.

**Table 3:** Correlation of treatment options with tumour control and overall survival.

	Total	Tumour control at 4–7months		Survival (No./%)	
Therapy combinations	No./%	No Lesion	Residual	Alive	Dead
1. EBRT alone	78(37.3)	65 (40.1)	13 (27.7)	49 (62.8)	29 (37.2)
1+ External boost	47(22.5)	28 (17.3)	19 (40.4)	24 (51.0)	23 (49.0)
1+ Brachy	18(8.6)	15 (9.3)	3 (6.4)	14 (77.8)	4 (22.2)
1+ chemo	52(24.9)	41(28.3)	11 (23.4)	45 (86.5)	7 (13.5)
1+ brachy + chemo	14(6.7)	13 (8.0)	1 (2.1)	13 (92.8)	1 (7.1)
Total/ p-value	209(100)	p<0.014		p<0.001	

Most patients received only the initial EBRT (37.8%), while 24.9% and 8.6% got additional chemotherapy and brachytherapy respectively. Only 6.7% of patients received combined treatment of EBRT, brachytherapy and chemotherapy. The kind of treatment options the patient received seemed to significantly affect tumour response and overall patient survival as shown here. On adjusting for age and HIV status through multivariate cox proportional hazards regression analysis and multinomial logistic regression, this statistical significance was maintained.

All patients had a pelvic bimanual examination to assess tumour response to treatment. Additional investigations done in this regard were as follows: 26.8% had ultrasound scans, 21.1% had X-rays, 16.2% had CT-scan, 1.0% had secondary biopsies, while only one patient had a radio-isotope examination (bone scan). None had MRI.

### Tumour Response

There was a significant association between the treatment options the patient received, the tumour response and subsequent overall survival (p<0.014 and 0.001 respectively) (Table 3). Patients who received optimal treatment of combined EBRT, brachytherapy and adjuvant chemotherapy had improved tumour control and better survival. Conversely, survival was significantly influenced by the observed tumour response to treatment when evaluated at 4–7 months following the initial 25 sessions of EBRT (P<0.001).

Although CCRT produced more cases of grade 3–4 overall acute toxicity than EBRT alone, the difference was not statistically significant. However, combined EBRT, brachytherapy and chemotherapy had significantly higher gastrointestinal grade 3–4 acute toxicity than EBRT alone (p<0.04) (Table 4). No deaths occurred directly due to acute treatment toxicity.

**Table 4:** Acute toxicity (grade 3–4) following various treatment modalities (N = 209).

Variable	All Patients	EBRT alone	EBRT	EBRT	EBRT + Brachy	P-value
			+ Chemo	+ Brachy	+ Chemo	
	(n = 209)	(n = 125/59.8%)	(n = 52/24.9%)	(n = 18/8.6%)	(n = 14/6.7%)	
Overall toxicity	73/34.9%	39/31.2%	19/36.5%	8/44.4%	7/50.0%	0.56
Gastro-intestinal	50/23.9%	26/20.8%	14/26.9%	4/22.2%	6/42.9%	0.04
Skin toxicity	34/16.3%	17/13.6%	9/17.3%	5/27.8%	3/21.4%	0.39
Genito-urinary	25/12.0%	11/9%	9/17%	3/16%	2/14%	0.18

Incidence of acute cute toxicity (grade 3–4) following various treatment modalities (N = 209). Although chemoradiation had higher cases of grade 3–4 overall toxicity than EBRT alone, the difference was not statistically significant. However, combined EBRT, brachytherapy and chemotherapy had significantly higher gastrointestinal grade 3–4 toxicity than EBRT alone (p<0.04).

Comparison of histological diagnosis versus disease progression indicated that, those with poorly differentiated SCC were almost twice as likely to progress (OR = 2.0 (0.8−4.9) and 2.5 times more likely to die than those with well differentiated SCC. Indeed overall patient survival was clearly influenced by both histological diagnosis (p<0.046) and FIGO disease stage (p<0.001) (Table 5).

**Table 5:** Correlation of FIGO staging and histological diagnosis with survival.

	Alive n/%	Dead n/%	P-value
**Staging**			
1A (2)	1 (50.0)	1 (50.0)	
1B (13)	10 (76.9)	3 (23.1)	
IIA (19)	16 (84.2)	3 (15.8)	
IIB (69)	59 (85.5)	10 (14.5)	0.001
IIIA (35)	21(60.0)	14 (40.0)	
IIIB (50)	33 (66.0)	17 (34.0)	
IV A (13)	4 (30.8)	9 (69.2)	
IV B (8)	1 (12.5)	7 (87.5)	
Total.	145 (69.4)	64 (30.6)	
**HPE (SCC) results**			
Well Differentiated	37 (27.6)	7 (12.3)	
Moderately	56 (41.8)	30 (52.6)	
Poorly	36 (26.9)	19 (33.3)	0.046
Keratinizing	2 (1.5)	1 (1.8)	
Large non-Keratinizing	3 (2.2)	0 (0.0)	
Total.	134 (70.2)	57 (29.8)	

Patients with poorly differentiated SCC were more than 2.5 times likely to die as compared to those with well differentiated SCC, while those with more advanced disease staging had as expected, higher death rates (p<0.46, and 0.001 respectively). Even after adjusting for age and HIV status using multivariate cox proportional hazards regression analysis and multinomial logistic regression, the disease stage was significantly associated with overall survival.

### Kaplan Meier Survival Curves

**Figure 2:**
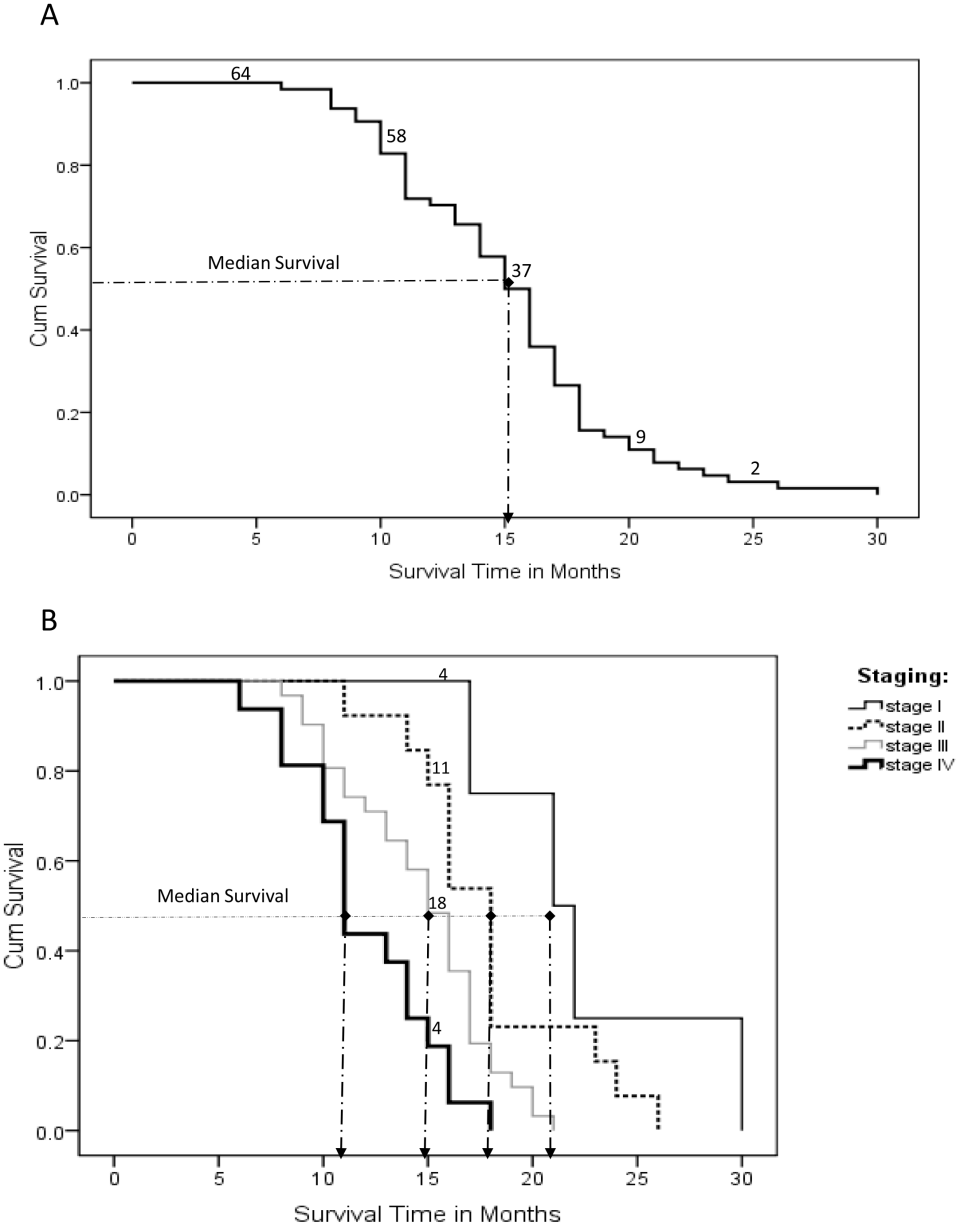
Kaplan Meir Survival Curves. (A). Overall median survival of 15.0 months; (B) Stratified median survival based on FIGO staging, whereby, stage I was 21 months with stages II, III, and IV having 18, 15 and 11 months respectively.

Among those who died, the mean time to death after the onset of treatment was 15.1 months while the median survival was 15.0 months as shown by Kaplan Meier curves (Figure 2). Median survival for FIGO Stage I was 21months while stages II, III, and IV were 18, 15 and 11 months respectively. At less than 20% the reported two year survival rate is very low.

## Discussion

The most important finding from this study was the low overall survival rates across all ICC FIGO stages among patients undergoing treatment at the main cancer referral centre in Kenya. To our knowledge, this is the first such investigation carried out in this geographical location. Another important finding was the low level of awareness of the importance of cervical cancer screening and equally disappointing were the low numbers of previous cervical smears carried out in the study population.

Our results demonstrate there is a significant association between the kind of treatment options that the patient received and overall survival (p<0.001). As expected, patients who received optimal combined therapies had better tumour control and improved overall survival. A number of women reported the use of traditional herbs although these were not standardized and there was no difference in treatment outcomes between those who used these and those who did not.

In spite of previous clinical trials which showed up to 30% improvement in overall survival of patients treated with CCRT [16], [17], [18], due to cost implications, relatively few patients actually received this treatment despite this being prescribed. Not surprisingly, those who did receive CCRT had better overall survival than those who received EBRT alone. In our study those patients who were given chemotherapy received mainly cisplatin and 5-fluorouracil since the addition of both these agents has been shown to significantly improve the survival rate of women with locally advanced ICC without increasing the rate of late treatment-related side effects [19]. However, it should be noted that our interpretations of treatment efficacy are limited by the low frequency of followed up patients, limited duration of follow-up and the small number of cases receiving adequate treatment. In a retrospective analysis of management outcomes of 3,892 cases of locally advanced ICC's in Chennai India it was shown that treatment with EBRT alone produced the lowest 5-year disease free survival (DFS) (37%). Surprisingly use of CCRT produced little improvement in DFS (41%) although inclusion of brachytherapy with EBRT significantly enhanced this (58% p<0.001). However, the combination of CCRT with brachytherapy resulted in the best DFS (69%), irrespective of disease stage. These findings clearly indicate that current best practice for locally-advanced ICC should be CCRT which includes brachytherapy [20] and there are many studies which support the benefits of CCRT for the management of ICC [21], [22], [23].

However, as we have reported, not all patients qualify for CCRT and similar findings were reported by McArdle and Kigula-Mugambe JB, (2007) who conducted a prospective study to assess the eligibility of Ugandan ICC patients for CCRT [24]. Following assessment, 47 patients (15.1%) were eligible for combined modality treatment whereas 190 (60.5%) were not and this could not be established in 77 cases (24.4%). The most frequently encountered exclusion criteria were hydronephrosis with impaired renal function and anaemia although a HB cut off point of 8 g/dL resulted in the exclusion of 55 (17.4%) patients with an additional 11 patients excluded by a limit of 10 g/dL.

It is significant that in the current study, only 36% of the women had ever heard of cervical cancer screening test and only 15% had actually ever had a pap smear of which 16% never received their results and all these women went on to develop ICC. Similar low levels of awareness have been previously reported in Kenya [25], [26] and other non-African locations [27] and it has been concluded that poor frequency of Pap screening is a primary factor in the development ICC [28]. Barriers to adequate screening reported from various African locations include; poor education, poor access to public health facilities, lack of knowledge concerning prevention and symptoms of cervical cancer, negligence, fear, embarrassment and finance [29], [30], [31]. These factors all contribute to the low Pap smear screening coverage in sub-Saharan Africa which ranges from 2.0% to 20.2% in urban areas and 0.4% to 14.0% in rural areas [32].

What is clear is that current screening strategies are ineffective at reaching the majority of the at-risk population in developing countries. Possible cost effective strategies to improve this situation could include activities such as: improving education and information, strengthening quality control procedures, addressing issues related to cultural beliefs, encouraging open discussion about women's needs and increasing the number of female care providers [29], [30], [31]. It is obvious there is a great need to educate women on the benefits of cervical cancer screening, to improve the uptake of Pap smear in these countries. Equally, it is crucial to create awareness among primary care practitioners in order to develop an effective referral pathway in addition to investing in health services to effectively diagnose and treat ICC in Sub Saharan Africa. There is clearly a need for primary practitioners to be aware of the aetiopathology of cervical cancer, it's preventable and curable nature and their role in the management of both pre-malignant and invasive disease. Increased training and empowerment of practitioners needs to be combined with provision of the necessary infrastructure to ensure one-stop screening and treatment where VIA or VILLI is used to inform the decision for immediate cryotherapy or LEEP. This should ensure cost-effective access to preventive and early treatment of premalignant cervical lesions. In addition there is also a need to establish an effective referral systems from primary health centres to regional/national centres for specialist management of invasive cancer. Furthermore, it is clear that increased investment in capacity building and infrastructure will facilitate decentralised management of ICC to regional centres which should alleviate the congestion at single national referral hospitals to reduce the waiting time between diagnosis to treatment.

Concerning the influence of ICC staging and histology, our results showed that, the majority of patients (80.5%) presented with advanced stage 2B or above (Figure 1) and is consistent with previous studies carried on in Africa where >80–90% of women present with late stage disease [33], [34]. However, this does not explain the low overall survival observed in the current study. For example, developed countries have reported 5 year overall ICC survival rates of approximately 68% [12], [35], [36]. Indeed 80–90% of women with stage I and 50–65% with stage II ICC are still living 5 years after treatment. Furthermore, 25 to 35% of women with stage III and 15% of those with stage IV cancer have >5 year survival [35]. Additionally, in the US, Brookfield et al (2009) found an overall median survival in 5367 ICC patients of 43 months. However, this was lower at 28.8 months in African Americans when compared to 47.1 months in Caucasians (p<0.001) [37]. In our study, the median survival was extremely low at 15.1 months. This clearly indicates that early detection is not the only reason for higher survival among patients in developed countries since improved treatment outcomes are seen across all FIGO stages of the disease. The explanation for this poor survival in African locations is not clear although it is possible this could be related to inaccurate clinical staging?

Clinical examination (CE) was the mainstay of assessing initial staging and response to treatment in our study. Although various investigations were requested to supplement CE, few patients could afford these additional tests which were: ultra-sound (27%), X-Ray (21%) and CT (6%). In our study no patient had MRI due lack of availability and high cost. Nevertheless, the correlation between CE and MRI has been reported as good in early stage disease although this worsens with advanced local disease [38]. Kodaira T, et al (2003) also found that MRI provides improved diagnostic information over FIGO stage for patients with bulky disease and yet this is still a good prognostic factor for patients with non-bulky disease (volume </ = 50 cc)[39]. However, for ICC patients selected for non-surgical treatment, radiological assessment of tumor size and lymph node status does provide valuable prognostic information over and above FIGO staging alone [40]. Thus it is possible that the poor treatment outcome reported in our study could have been due to ‘under-staging’ where women had more advanced disease than was diagnosed. Undoubtedly, another contributory factor was that it took an average of 2–3 months from diagnosis to commencement of treatment for most cases. Indeed, 21% were initiated within one month, 44% within 2 months, 31% within 3 months while 4% did not commence treatment until the 4^th^ month after diagnosis. The reasons for these ranged from socio-economics, difficulties with travelling, inability to gain admission to crowded hospital oncology wards and queues of patients awaiting treatment with the single radiotherapy machine at KNH.

Although lymph node (LN) status is undeniably the most important prognostic indicator, FIGO staging is most often used in low resource settings [41]. However, this has an error rate of approximately 25% in stage I and II disease and 65%–90% in stage III and IV disease [41] which undoubtedly contributes to the differences in survival observed between patients who are ascribed the same disease stage. For example, in stage IB disease, the survival rate is 85%–95% for patients with negative nodes at surgery and 45%–55% for those with positive nodes [42]. Thus it is clear that these errors are most likely due to under-staging since it is difficult to accurately measure the extra-cervical spread of disease [41]. This has prompted the increased use of CT and MRI in the developed world. Even though LN involvement is the single most important prognostic factor for ICC [40], in resource poor countries such as Kenya, CE is still most commonly used. In order to improve this, it may be possible to change referral procedures to decrease the long waiting time between diagnosis and commencement of treatment since this may reduce the risk of progression of ICC to less treatable stages.

Concerning treatment related toxicity, our study showed that although CCRT had higher cases of grade 3–4 overall toxicity than EBRT alone, the difference was not statistically significant. However, combined CCRT and brachytherapy, not surprisingly, had significantly higher gastrointestinal grade 3–4 toxicity than EBRT alone (p<0.04). There was no significant difference in genitourinary and skin toxicities from any of the treatment combinations. Therefore use of CCRT for the treatment of locally advanced ICC is feasible and produced acceptable toxicity. EBRT was well tolerated and all patients completed the whole course. These findings are consistent with previous studies which showed that non-haematological grade 3 and 4 toxicities were considerably more common in the CCRT groups than the EBRT groups [43]. Furthermore gastrointestinal toxicity was twice as common in women randomised to CCRT (*P*<0.001), with 8% of these patients suffering severe or life threatening adverse events. In the current study, we did not assess haematological toxicity.

Our results showed there was a high incidence of anaemia in our patients with 37% requiring blood transfusion during the course of their treatment. Given that tumour hypoxia is a well known predictor of response to RT treatment this scenario could also have contributed to the poor outcomes. Iron deficiency and tumor bleeding are common causes of anemia in ICC [44] which are treated with either transfusion and/or erythropoietin prior to treatment. Indeed many investigators have demonstrated tumour hypoxia has a negative impact on the ability of RT to influence loco-regional control of tumours [45], [46], [47] and hypoxic tumours cells are also known to be more resistant to chemotherapy. Thus, hypoxia plays a key role in tumour prognosis since it enhances therapy resistance and also promotes the development of more malignant phenotypes [48].

Concerning the impact of HIV status on survival from ICC, in our patient cohort, 27.5% of those tested were positive for HIV and had poorer survival when compared to HIV-ve women and yet, surprisingly, this did not achieve statistical significance. A similar investigation carried out in the same institution between 1989 and 1998 found a HIV prevalence of 15% and concluded that the prevalence in ICC patients was comparable to that found in the general population at the time.[49]. However, in the USA, cervical cancer has been reported to be the most common malignancy among women with AIDS [50]. Indeed based on data from the CDC, the incidence of cervical cancer is approximately 900 per 100,000 in women with AIDS, as compared with about 10 per 100,000 in the general population [51]. These data indicate that not only are women with HIV more likely to develop ICC, but also the course of the disease may be worsened by the presence of the virus. Moreover, HIV-positive women with ICC are more likely to be diagnosed at a later stage, have a poorer response to therapy and have a higher rates of recurrence than HIV negative women [11]. Furthermore, ICC is known to progress rapidly in HIV positive women [52], [53]. In addition previous work has shown that HIV infection is also associated with increased risk of multisystem radiation-related toxicity; treatment interruptions and locoregional failure following EBRT [11]. In our study, since 46.8% of women had unknown HIV status it is possible that some of these could have been HIV+ve which could contribute to the observed poor outcomes. Interestingly, current HIV prevalence in Kenya is 8.0% among women in the general population [54].

Obtaining follow up data is a massive problem in African locations and, of the initial 355 patients enrolled in our study, 146 (41.1%) patients were lost to follow-up which is typical for comparable geographical locations. It is most likely that financial considerations were the driving force behind this level of attrition since a study carried out in Nigeria showed that of 95 ICC patients referred for RT, only 19% (n = 18) actually underwent the procedure whilst the remaining 81% (n = 77) did not attend due to lack of funds [55]. It is also very significant that all the patients that underwent RT were in the upper social class and used approximately 30% of their annual income for the treatment. Thus, our study further serves to emphasize the difficulties encountered in ensuring patients receive adequate and timely diagnosis, staging, treatment and follow-up in this area of the world.

It is very clear that the magnitude of the cancer burden in many low resource countries is poorly understood owing to lack of monitoring systems to assess cancer incidence, survival and mortality. Therefore the current study aims to raise awareness of the scale of this problem for ICC in Kenya. Cancer diagnosis and care services are woefully inadequate in low- and middle-income countries with late-stage presentation being a common feature that results in less potential for cure and more need for symptom management [56]. And yet palliative care services are also grossly inadequate in such countries, with many cancer patients dying unnecessarily painful deaths.

In our study very few women received the optimal recommended treatment and therefore it is perhaps not surprising that the outcomes are so poor even for women with stage 1 disease. What are the barriers to providing full treatment? The main obstacle is that patients have to pay and simply can not afford it. Since the health system funding in Kenya is unlikely to change, it is suggested that implementation of a workable screening programme combined with a system for treating women with early-stage abnormal smears should be considered as a viable option. Clearly prevention is far preferable and much cheaper than the previously discussed treatment options for ICC. However, this is not straightforward! Any type of screening service will have to be quality controlled, there must be adequate coverage and this must be accompanied by an educational programme to encourage women to attend. Cultural barriers, erroneous beliefs and misconceptions must be addressed which are common problems in many developing nations. What factors has the present study identified which could improve survival of women who currently develop ICC in Kenya? It is very clear that there is a great need for a standardized system of follow-up which focuses mainly on the actual care that women are getting. One suggestion would be to standardise diagnostic and follow-up investigations making these simpler which would cut costs and potentially reduce the lead-time from diagnosis to commencement of treatment. For example, given that so few women are able to get the recommended pre-treatment imaging (CT/MRI), it may prove more effective for all women to get EUA with biopsy and X-ray to check for hydronephrosis which will then form the basis of treatment decisions. Since the majority of women are self-funding and have limited resources it makes sense to use these monies for the most important treatment-associated costs. This, should, in turn, increase the numbers able to complete the treatment program.

Unlike the situation in the majority of developed countries it is important to identify and prioritize which resources address healthcare needs most effectively and to consider alternative approaches in countries like Kenya. Therefore, given the challenges in implementing organised screening programs, the value of implementing HPV vaccination (i.e in 9–13 year old girls) cannot be over-emphasized since this will eventually build a cohort of women at very low risk of cervical cancer. Prophylactic HPV vaccines as a primary intervention against ICC may be used as part of the WHO's widespread Expanded Program of Immunization (EPI) i.e. childhood vaccination program in developing countries [57]. Additionally, there is now an opportunity to implement this approach in Sub Saharan Africa with the assistance of GAVI which will clearly help establish the necessary capacity and infrastructure. Indeed such partnerships will ensure increased access to immunization for children in poorer countries where cost is obviously a major issue although, in the long term, this approach may prove to be the most cost-effective.

In summary, more studies of this type are needed to strengthen ICC as a public health priority in Sub Saharan Africa since publicizing the extent of this problem may help to drive implementation of some of the potentially life saving changes discussed.
